# Supplementary material for: The practice of gender and protection mainstreaming in health response in humanitarian crisis - A case study from the refugee camps in Cox’s Bazar, Bangladesh
Source: PLoS One. 2025 Jul 1;20(7):e0310878. doi: 10.1371/journal.pone.0310878 (PMC12212524; doi:10.1371/journal.pone.0310878)
Supplement: S1 File — (DOCX) [file pone.0310878.s001.docx]

**Guiding questions for key informant interview**

1. Would you kindly mention and explain existing practices on gender and protection mainstreaming in health response in Rohingya refugee camps? Kindly share your experiences in different health responses, e.g. primary health care, community health, epidemic and disaster response.
2. Please comment on how effective these practices are in achieving gender and protection mainstreaming in health response. Why do/don’t you think the practices are effective? You can explain the effectiveness of the practices, guidelines and/or tools by elaborating how these are performing in terms of a) implementing planned interventions; b) achieving the objectives; c) accessibility, availability and affordability of the service/interventions; d) acceptability of the interventions; e) safety; and f) competence.
3. Please comment on how efficient these practices are in achieving gender and protection mainstreaming in health response. Why do/don’t you think the practices are effective? You can explain the efficiency of the practice, guideline and/or tools in terms of a) time efficiency; b) cost effectiveness; c) technical efficiency; and d) relevance and e) sustainability
4. Please comment on how satisfactory the practices are in in achieving gender and protection mainstreaming in health response. Why do/don’t you think the practices are satisfactory? You can explain the satisfaction of the practice, guideline and/or tools in terms of a) improving people’s satisfaction; b) promoting trust; c) reducing inequality; d)
5. Would you kindly mention and explain available guidelines and tools on gender and protection mainstreaming in health response that are used or proposed for use in Rohingya refugee camps?
6. Please provide your views on the mentioned tools and guidelines on protection and gender mainstreaming in terms of a) competence; b) achieving objectives; c) technical, time and cost efficiency; d) relevance; e) gender and inclusiveness analysis; f) ease of understanding and h) indicators that influence individual or population conditions.
7. From your view and experiences, what are the gaps and challenges in terms of protection and gender mainstreaming in generally emergency health response, particularly in Rohingya refugee camps?
8. From the discussion so far, which of the practices you would identify as best practices. Do you think these practices can be replicable in other current and future local and global humanitarian crises?
9. Do you have some recommendations to improve the gender and protection mainstreaming in health response generally in humanitarian context, specifically in Rohingya refugee camps, addressing the gaps and challenges you have mentioned?
10. Do you want to share any other thoughts or information that you might think helpful for this research?
