# Supplementary material for: The practice of gender and protection mainstreaming in health response in humanitarian crisis - A case study from the refugee camps in Cox’s Bazar, Bangladesh
Source: PLoS One. 2025 Jul 1;20(7):e0310878. doi: 10.1371/journal.pone.0310878 (PMC12212524; doi:10.1371/journal.pone.0310878)
Supplement: Attachment — Submitted filename: comments peer review.docx [file pone.0310878.s003.docx]

Peer review PLOS ONE

*5. Review Comments to the Author

Please use the space provided to explain your answers to the questions above. You may also include additional comments for the author, including concerns about dual publication, research ethics, or publication ethics. (Please upload your review as an attachment if it exceeds 20,000 characters) (Limit 200 to 20000 Characters)

I want to thank the author for this interesting manuscript. It is certainly insightful and supportive to the gender mainstreaming and humanitarian response in camp/emergency settings and beyond. I particularly appreciated the concise discussion, conclusions and recommendations provided – which will certainly be valuable to actors in Cox’s Bazar and similar contexts.

I am less familiar with the method chosen, yet did my best to contribute to improvement of the work.

Re question 3, I am uncertain if PLOS requires qualitative data to be shared, which I would doubt. I clicked NO, as no data set is made available, yet I assume this is not needed in this case.

Re question 4, I would advise for a thorough review and editing process, reducing repetition and increasing concise and clear language. Particularly the result section could be improved. The discussion and conclusion sections were really good.

I am providing very detailed comments with the aim to improve the manuscript and its contribution to the field.

Further proof reading and support with condensing could be useful. At times sentences are very long, and could be broken up into 2 or more to aid understanding.

**Introduction**

Persons with disability and older persons often face discrimination based on their age and disability. -> switch age and disability at the end to match the order of the start of the sentence.

To meet the demand currently, 80 health partners are providing primary and secondary healthcare services in the camps through 93 health posts, 43 Primary Health Care centres (PHCs), Field Hospitals and a network of 1300 community healthcare workers (CHW) (13). => currently should be placed before demand, to say “to meet the current demand”; or after “are”, to say “are [currently] providing” if the emphasis is that the stated numbers are based on the current state (and of course constantly will shift)

Adding an s to the following terms (if I am reading this right) ... staffing [recruiting/ensuring?] representative[s] of gender, ethnic and economic differences, provision of reproductive and obstetric health services, provision of private breastfeeding space[s] ...

IASC guidelines => writing these abbreviations out at first time

Consistency in using psychosocial or psycho-social

... in evidence regarding the effectiveness of inclusion efforts, use of disability and age-disaggregated data over 60 years, cost and benefits of inclusion strategies, and the intersection of disability and older age (8,31). => I assume you may want to include gender in the last part of the sentence, as you are looking at the intersection of gender, disability and older age – otherwise I do not full understand the message of this paragraph, which is also a bit oddly placed between the previous and the subsequent

However, except in the area of SRH services, minimal studies have been [are? Or have been made?] available that have generated evidence on the uptake, practicality, and effectiveness of those interventions.

Given that the protection and gender mainstreaming is a complex phenomenon which involves awareness, actions, behaviour and capacity of a broad spectrum of stakeholders, including policymakers, humanitarian workers and the community, a qualitative approach was chosen to gain an understanding of what interventions in gender and protection mainstreaming are being taken, how practical or effective they are, what is going well and what is not, and how we can further improve the system. => advise to brake into at least 2 sentences

Further, a case study as a qualitative study type is well suited to... => Further, a qualitative case study is well suited to... [may be a shorter way of saying the same thing]

Since no study is available that gives a comprehensive picture of the practicality of gender and protection mainstreaming in health response in the humanitarian context, a case study of the refugee context in Cox’s Bazar was chosen to gain a comprehensive understanding of this complex phenomenon. => sentence could be condensed and avoid repetition

**Research philosophy/methods**

I am not so familiar with qualitative work, and respective journal requirements. I am therefore not sure if it is common to have subtitles of “research philosophy” and “research setting”, rather methods/approach and setting.

WASH => should be written out the first time it is used, even if commonly understood within the humanitarian sector

...it was found that the total sample size of 12 was enough for this study. => sufficient would be a more appropriate word rather than enough, in my experience of journal writing

On the “Data collection instrument” section: In my experience interviews are rather conducted with topics guides, rather than questionnaires; questionnaires tend to be used for quantitative data collection – yet this might be my lack of knowledge within the qualitative methods field

Microsoft Team => Microsoft Teams

Who/how were the interviews transcribed? By teams or by a person? This is not clear. I see this is covered in analysis, I think this can rather fit in process above

“In the case of online interviews, the recording was done by the Microsoft team, and after transcription writing, the recordings were permanently deleted.” => delete “writing” in this sentence

What about translation? As some interviews were conducted in Bangla

Data analysis

This section may need to be re-written/re-ordered, as particularly the first 3 sentences do not flow.

Also the sentences of “Thematic analysis was done for the data collected through in-depth interviews since it is the appropriate method for the analysis of experiences, thoughts and behaviours (36)” is confusing, and I think can be shortened to -> The in-depth interviewed were analysed using thematic analysis, as the methods is appropriate for exploring experiences, thoughts and behaviours – or something along those lines

university-provided P-drive => is not clear to an outsider, maybe you mean to say password-protected server?

**Results**

Although introduced in the first paragraph “Later, we presented the findings from the in-depth interviews...”, I perceived the switch from report/guideline synthesis based on the reviewed literature to the quotes of from interviews as abrupt. I would advice some (re)introduction when findings from the interviews are presented.

From my experience of reporting on mixed methods (quant and qual), these should either be combined, and then presenting per theme; or into clear sections of e.g. first quant and then qual. I assume the same could be done with the literature insights and the interviews – either they should be integrated and presented together per theme; or separated – or, as said above, some “taking the reader by the hand” is needed when moving to another section.

“It has been found in the health sector quarterly monitoring report that all health facilities (100%, n=136) have a minimum of a female medical professional in the functioning hours of the facility (44).” => this could be formulated with more clarity, who/how/where this has been found, e.g. a report on XX / a review on XX showed that XY. I find “it has been found” vague and would prefer clarity

“Participants expressed that having female staff in the health facility allows women patients/clients to access health care from the facility comfortably; they feel more comfortable speaking and taking part in physical examinations.” => you could cut “from the facility”, and add more elegantly and avoiding repetition “including speaking (up) during and taking part in physical examinations”.

“According to the number of consultations in a medical facility, as several participants mentioned, women use the facilities more frequently than males in the Cox's Bazar context.” => this is confusing, is this based on the number of consultations or based on the reports of several interview participants -> this sentence might need reordering and clarity

Again I would advise to condense “This is a fact for leadership positions as well. Women have less representation in leading positions, like health facility managers or coordinators.” => I would advise something along the lines of: Particularly in leadership positions, such as facility manager or coordinator roles, female staff is scare.

“During the study, “ => do you mean, at the time of the study?

CMR = abbreviation should be clarified at first time use

“Participants also opined that there are only a few training initiatives are undertaken for...” => please check grammar

Even if in a quote, I would advice to use [...] brackets and clarify the meaning of ETAT

PHC = abbreviation should be clarified at first time use

“... stretchers (58% health posts and 86% PHCs), and Crutch...” => delete “and” to make this consistent in your sentence, and make crutches plural and lower case

“...to increase the accessibility of persons with disability and older persons.” => to increase accessibility/ access for persons...

“Although the protection mainstreaming guideline[s?] recommends that latrine design account[s] for children, this is barely taken into account [considered?] during latrine installation.” => please check grammar and avoid repetition of wording if possible, examples provided in breakers as an idea

“... and engineers in 2019 on this.”=> check grammar, it would flow better as “... on this in 2019.”

“A few agencies have some space for children to play or art.”=> create/do art or draw? art is not a verb, please also edit in line 857

Check paragraph of “Targeted interventions toward [for?] vulnerable groups” => some errors in punctuation and spacing, and please consistently use “persons with disabilities” or “people with a disability”

“This can be linked to the fact that 56% of persons...” => this sounds bumpy and confusing; I advise rather something like “this is underlined by / also reported by and assessment conducted by...”

IOM = abbreviation should be clarified at first time use

From my understanding of qualitative work, every quote should have clarity on who stated this, for example with brackets after the quote e.g. (health care specialist), (female expert on GVB) or something alike. Given your field, it might be useful to show the gender of the participant, if allowed and ensuring anonymity

FDMN = abbreviation should be clarified at first time use

Make consistent use of % instead of using the word per cent, unless the first word of a sentence; also numbers above twelve should be written out in numbers, unless the first word of a sentence -> please check this throughout your manuscript

“Frequent staff turnover is another challenge requiring organising training for the newly joined staff.” = please review grammar and flow

“Participants mentioned a good practice that some health facilities are facilitating community health facility support groups with representation from different gender and ages, persons with disability and community leaders. This group owns the health facility and provides feedback through periodic meetings with the facility managers.” => I am not sure I understand this, which group owns the health facility? The community health facility support group that is consulted, or do you mean to say that the group informs the facility? => please clarify

Is there an error in the quote or typo? “*Have we considered* *how we will reach out to those messages who have difficulty in speech and hearing” ?* => messengers? Or should the word ; messages’ be deleted, please clarify and it may be possible to use ... in between instead

OPD = abbreviation should be clarified at first time use

“However, experts have opined that this tool cannot be used for children below two years of age.” => is this the main disadvantage and reasons for it not being used? I assume even in high-income countries some disabilities may not be noticed until after the age of 2 years, so I find this comment confusing or the argument may need strengthening or clarity

MMT = should be put into brackets the first time it is written out, to help the reader, please edit

“... include all adults (>18 years)” => I think you want this to say ≥ 18, as otherwise you are not including the 18 year olds, but only those older than 18.

**Discussion**

I really like the discussion, it flows nicer than the results, and is more concise and clear. It provides good synthesis of the collected data and situates this in the literature.

“Our study found that although it is in principle to engage different layers of the community ...” => please check grammar line 897, I assume you mean “it is a principle to..” but then I would ask by whom, I assume the sector?

“Our study echoes the finding of An After Action Review following a massive fire incident in 2021 that a protection focal person from the protection sector in MMT is a best practice for protection mainstreaming in emergency health response...”=> I do not fully understand what this report found, could this be clarified? What did the focal point do? Can we learn from this situation for others?

**Conclusion and Recommendations**

“Although the research addressed gender and protection mainstreaming at the local level, many of the findings can be generalised to other similar humanitarian contexts; consequently, the global health and protection clusters..”. => I recommend to break this up and simply start a new sentence with “Consequently, ...”
