# Supplementary material for: The practice of gender and protection mainstreaming in health response in humanitarian crisis - A case study from the refugee camps in Cox’s Bazar, Bangladesh
Source: PLoS One. 2025 Jul 1;20(7):e0310878. doi: 10.1371/journal.pone.0310878 (PMC12212524; doi:10.1371/journal.pone.0310878)
Supplement: Attachment — Submitted filename: Response to Reviewers.docx [file pone.0310878.s005.docx]

**Manuscript ID:** PONE-D-24-39518

**Title:** The Practice of Gender and Protection Mainstreaming in Health Response in Humanitarian Crisis – A Case Study from the Refugee Camps in Cox’s Bazar, Bangladesh

**Dear Respected Concerns,**

Please accept our sincere gratitude; we appreciate the detailed and constructive feedback provided throughout the peer review process. It was instrumental in strengthening the clarity, rigour, and coherence of our manuscript. We carefully addressed each point and made the necessary adjustments accordingly. We have provided detailed responses to all comments raised by the reviewers. We have added the qualitative dataset (thematic analysis) - as supplementary information. We removed sensitive information (e.g. agency name) that could identify the participant.

**Response to the Academic Editor’s Comments**

**Comment 1:** Ensure that the manuscript meets PLOS ONE’s formatting guidelines.

**Response:** We have reviewed and updated our manuscript according to PLOS ONE’s style requirements. The file naming convention was aligned with journal standards, and the manuscript was structured according to the specified formatting templates.

**Comment 2:** Confirm whether the submission contains all raw data required to replicate the study findings.

**Response:** We confirm that all relevant data supporting our findings are included within the manuscript and its supplementary materials. We have ensured that the values utilised for generating key statistics, graphs, and supporting analyses are accessible.

**Comment 3:** Ensure that the ethics statement is placed within the Methods section only.

**Response:** The ethics statement has been relocated to the Methods section, and all duplicate references have been eliminated to adhere to the journal's guidelines.

**Response to Reviewer 1’s Comments**

**Comment 1:** Provide more details on the process of obtaining consent and ensuring participant confidentiality and anonymity.

**Response:** We have expanded the Methods section to clearly explain how informed consent was obtained from participants and the steps taken to maintain confidentiality and anonymity, including secure data storage and anonymized reporting.

**Comment 2:** Was emotional support available for participants in case of distress?

**Response:** We have clarified that participants were informed of available psychological support services in case they experienced distress while participating in the study. This information was conveyed to them via the consent process and accompanying letters.

**Comment 3:** Statistical power considerations should be discussed.

**Response:** Although our study is qualitative, we have recognised the importance of statistical power in relation to qualitative sample adequacy and have explained the appropriateness of our sample size for achieving thematic saturation.

**Comment 4:** Consider discussing deviations from normal distribution in the data analysis section.

**Response:** This qualitative study does not involve variables that necessitate normality testing. However, we have provided further methodological justification for the analytical framework.

**Comment 5:** The discussion is too descriptive and lacks broader implications.

**Response:** We have refined the Discussion section to move beyond summarization and incorporate a deeper analysis of implications for gender mainstreaming and protection strategies. New references have been incorporated in each section to enhance the argument.

**Comment 6:** Ensure that the abstract accurately summarizes the revised manuscript.

**Response:** The abstract has been revised to ensure consistency with the updated manuscript. It now provides a concise and clear summary of the study’s key contributions.

**Response to Reviewer 2’s Comments**

**Comment 1:** The title and study design need better alignment. Why is this considered a case study?

**Response:** We have clarified the justification for using a case study approach, referencing key methodological sources. Given the specificity of the refugee camp setting, our study follows a case study methodology rather than a general qualitative approach. Methods section has been updated with this clarification.

**Comment 2:** The term “Gender and Protection Mainstreaming” may be unfamiliar; consider alternative wording.

**Response:** We have included a formal definition from sectoral and global cluster guidelines to ensure clarity for a broader audience. We have preserved the original terminology to ensure consistency with international humanitarian frameworks.

**Comment 3:** The methods section lacks details about data analysis and potential bias.

**Response:** We have expanded the Methods section to specify participant selection, data analysis techniques, and measures taken to reduce bias, including reflexivity practices and triangulation techniques. A limitation related to bias has been incorporated into the Limitations section.

**Comment 4:** The Results section needs better participant details and demographic information.

**Response:** We have provided additional details about participant demographics, roles, and rationale for selection in the results section.

**Comment 5:** The conclusion should be reframed to better reflect the study’s findings without overgeneralization.

**Response:** Conclusion has been revised to reflect the study's contributions, avoiding overstatements and emphasising the relevance of findings to humanitarian contexts.

**Comment 6:** The manuscript lacks clear definitions and structure in the introduction and background.

**Response:** The Introduction has been revised to clearly define key concepts, including gender and protection mainstreaming. Coherence between the background and objectives sections has been enhanced.

**Response to Reviewer 3’s Comments**

**Comment 1:** Improve sentence structure and clarity in various sections, particularly the Results.

**Response:** We have conducted a thorough language review and proofreading process to ensure clarity and conciseness throughout the manuscript. Sentences have been shortened for improved readability, and redundancies have been eliminated.

**Comment 2:** Define all abbreviations at their first use.

**Response:** Abbreviations such as IASC, WASH, PHC, OPD, MMT, FDMN have been written out in full upon their first mention.

**Comment 3:** The transition between the literature synthesis and interview findings is abrupt.

**Response:** We have improved transitions between sections, ensuring that the shift from literature findings to qualitative insights is smoother and more intuitive.

**Comment 4:** Improve clarity in some quoted excerpts.

**Response:** We have revised quotes for clarity while maintaining their original meaning. Bracketed clarifications have been included where necessary to enhance comprehension.

**Comment 5:** Ensure consistency in numerical formatting and terminology.

**Response:** We have standardized numerical representations (e.g., using % instead of "percent") and ensured consistent terminology use throughout the manuscript.

**Comment 6:** Improve grammar and wording in specific sentences.

**Response:** All suggested grammatical revisions and rewordings have been implemented. This involves revising unclear sentences to enhance readability.

**Summary of Major Revisions**

- Manuscript formatting has been revised in accordance with PLOS ONE guidelines.
- The ethics statement has been relocated to the Methods section.
- Clarified the methodology of the case study and supplied relevant references.
- Enhanced definitions and terminology for improved clarity.
- Enhanced the methods and limitations sections to thoroughly examine data analysis and potential biases.
- Enhanced participant details and demographics in the Results section.
- Streamlined discourse to enhance analytical depth and incorporate additional references.
- The conclusion was revised to align with the findings while avoiding overgeneralisation.
  Conducted a comprehensive language review to enhance clarity, grammar, and structure.
- Maintained uniformity in abbreviations, terminology, and formatting.
- Qualitative dataset (thematic analysis) added as supplementary information.
- Removed sensitive information (e.g. agency name) which could identify the participant.

We greatly appreciate the reviewers’ insightful feedback, which has significantly enhanced our manuscript. We hope that our revisions meet the expectations of the editorial team and reviewers. We hope this will now resolve the requirement. We remain open to further feedback and look forward to the possibility of publication in PLOS ONE.

Thank you for your time and consideration.

Best regards,

**Charls Erik Halder**

MBBS, MPH, MSc (Global Health)

Global Public Health Specialist

Chattogram, Bangladesh

Email: [icyerik@gmail.com](mailto:icyerik@gmail.com)
